# Thromboembolic Disease in Haemophilic Patients Undergoing Major Orthopaedic Surgery: Is Thromboprophylaxis Mandatory?

**DOI:** 10.3390/diagnostics13010013

**Published:** 2022-12-21

**Authors:** Oana Viola Badulescu, Iris Bararu Bojan, Minerva Codruta Badescu, Nina Filip, Alina Chelsău, Manuela Ciocoiu, Maria Vladeanu, Alexandru Filip, Norin Forna, Mihnea Theodor Sirbu, Carmen Ungureanu, Paul-Dan Sîrbu

**Affiliations:** 1 Department of Pathophysiology, Morpho-Functional Sciences (II), Faculty of Medicine, “Grigore T. Popa” University of Medicine and Pharmacy, 700115 Iasi, Romania; 2 Department of Internal Medicine, Faculty of Medicine, “Grigore T. Popa” University of Medicine and Pharmacy, 700115 Iasi, Romania; 3 Department of Biochemistry, Morpho-Functional Sciences (II), Faculty of Medicine, “Grigore T. Popa” University of Medicine and Pharmacy, 700115 Iasi, Romania; 4 Institute of Cardiovascular Diseases, G.I.M. Georgescu, 700503 Iasi, Romania; 5 Department of Orthopedics and Traumatology, Surgical Science (II), Faculty of Medicine, “Grigore T. Popa” University of Medicine and Pharmacy, 700115 Iasi, Romania; 6 Department Morpho-Functional Sciences (I), Faculty of Medicine, “Grigore T. Popa” University of Medicine and Pharmacy, 700115 Iasi, Romania

**Keywords:** haemophilia, thromboprophylaxis, clotting factors

## Abstract

Haemophilia is a rare genetic disorder, that results from various degrees of deficiency of coagulation factor VIII (haemophilia A), or factor IX (haemophilia B), with an X-linked transmission. The patients affected are in the majority of cases males (who inherit the affected X-chromosome from the maternal side), with rare cases of females with haemophilia (FVIII or FIX < 40 IU/dL), situations in which both X-chromosomes are affected, or one is affected, and the other one is inactive (known as carrier). The hypocoagulable state due to the deficiency of clotting factors, manifests as an excessive, recurrent tendency to bleeding, which positively correlates with plasmatic levels. Severe haemophilia results in hemarthrosis, although recent data have shown that moderate or even mild disease can lead to joint bleeding. Recurrent episodes of haemorrhages, usually affecting large joints such as knees, elbows, or ankles, lead to joint remodelling and subsequent haemophilic arthropathy, which may require arthroplasty as a last therapeutic option. Orthopaedic patients have the highest risk among all for deep vein thrombosis (DVT) and venous thromboembolism (VTE) with morbid and potentially fatal consequences. While for the rest of the population thromboprophylaxis in orthopaedic surgery is efficient, relatively safe, and widely used, for patients with haemophilia who are considered to have a low thromboembolic risk, there is great controversy. The great heterogeneity of this particular population, and the lack of clinical trials, with only case reports or observational studies, makes thromboprophylaxis in major orthopaedic surgery a tool to be used by every clinician based on experience and case particularities. This review aims to briefly summarise the latest clinical data and to offer an insight into the current recommendations that readers would find useful in daily practice.

## 1. Introduction

Haemophilia is a rare genetic disorder that results from various degrees of deficiency of coagulation factor VIII (haemophilia A), or factor IX (haemophilia B), with an X-linked transmission. The patients affected are in the majority of cases males (who inherit the affected X-chromosome from the maternal side) with rare cases of females with haemophilia (FVIII or FIX < 40 IU/dL), situations in which both X-chromosomes are affected, or one is affected, and the other one is inactive (known as a carrier) [1]. There are significant prevalence differences between the two forms, as haemophilia A accounts for 80–85% of all cases, and haemophilia B for 15–20%. The diagnosis is based on factor assay to demonstrate the deficiency, and plasmatic levels can classify the disease in three forms: mild (clotting factor level 5–40 IU/dL, or 5–<40% of normal), moderate (clotting factor level 1–5 IU/dL, or 1–5% of normal), and severe (clotting factor level < 1 IU/dL, or <1% of normal) [1] (Table 1).

The hypocoagulable state due to the deficiency of clotting factors manifests as an excessive, recurrent tendency to bleeding, which positively correlates with plasmatic levels. Serious bleeding episodes occur at the joint level (leading to hemarthrosis), in 70–80% of cases, deep compartments of muscles in 10–20% of cases (iliopsoas, calf, and forearm), along with haemorrhagic events in mucous membranes of the mouth, nose, and genitourinary tract. In less than 5% of cases, life-threatening haemorrhages occur in the gastrointestinal tract, neck/throat, or intracranial [1].

Severe haemophilia results in hemarthrosis, although recent data have shown that moderate or even mild disease, can lead to joint bleeding [2]. There is evidence supporting the fact that even a single episode of bleeding can initiate the inflammatory process that leads to synovial thickening and irreversible angiogenesis, predisposing to recurrent bleeding [3]. These episodes, usually affecting large joints as knees, elbows, or ankles, lead to joint remodelling and subsequent haemophilic arthropathy, which may require arthroplasty [2] as a last therapeutic option. Moreover, patients with haemophilia likely require revision surgery after a first intervention.

Orthopaedic patients have the highest risk among all for deep vein thrombosis (DVT) and venous thromboembolism (VTE), with morbid and potentially fatal consequences [4]. The incidence of deep vein thrombosis ranges from 40% to 60% [5] in the absence of anticoagulation therapy. A patient in need of an orthopaedic surgery fulfils all the three criteria described by Virchow in 1884 [5,6] for a thrombotic event to take place: vascular endothelial damage, stasis of blood flow, and hypercoagulability. The immobilisation and bed rest, along with the usage of tourniquets, determine blood venous stasis. Endothelial vascular damage is present during surgical manipulation of the limb. Thromboplastic agents increase their level due to trauma, and the usage of polymethylmethacrylate (PMMA) bone cement leads to hypercoagulability [5]. In this prothrombotic milieu, venous thromboembolism prophylaxis is mandatory, and the current standard of care, with major guidelines from international societies stating the correct doses and duration of treatment [6]. A recent meta-analysis from 2021 including 25 studies [7], and a total of 40,438 patients, compared the efficiency of novel oral anticoagulants (apixaban, edoxaban, rivaroxaban, and dabigatran) (NOAC), with low molecular weight heparin (LMWH) in preventing venous thromboembolism in the general population. The study demonstrated a significant reduction in major VTE rate when using NOAC compared with LMWH (RR 0.33; 95% CI: 0.20–0.53; *p* < 0.01, I^2^ = 57%). A subgroup analysis by type of orthopaedic surgery (total hip arthroplasty versus total knee arthroplasty), demonstrated better results when using NOAC in total hip arthroplasty, compared with LMWH (RR 0.99; 95% CI: 0.77–1.27; *p* = 0.92; I^2^ = 4%). Major bleeding was also lower in NOAC patients. The study only emphasizes that prevention of embolic events with oral anticoagulant agents is associated with better efficacy outcomes and no significant differences in major bleeding and all-cause mortality.

**Figure 1:**
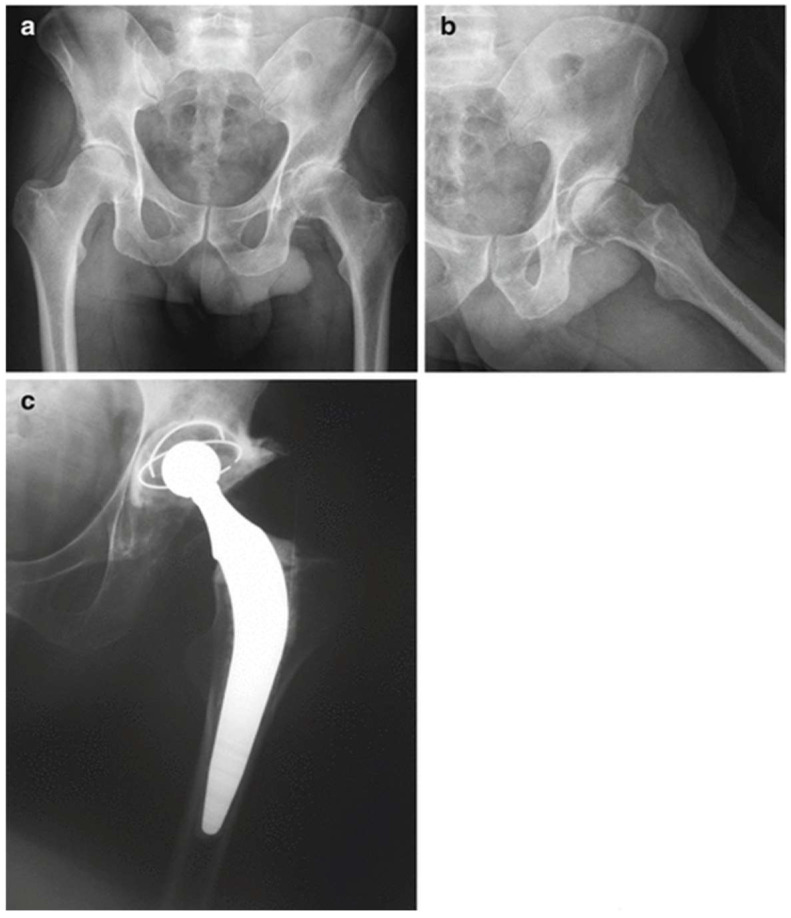
Total hip replacement (THR) in a haemophilia patient performed without using pharmacological thromboprophylaxis. Anteroposterior preoperative radiograph (**a**). Lateral preoperative view (**b**). Postoperative radiograph (**c**).

While for the rest of the population thromboprophylaxis in orthopaedic surgery is efficient, relatively safe, and widely used, for patients with haemophilia who are considered to have a low thromboembolic risk there is great controversy. The great heterogeneity of this particular population, and the lack of clinical trials with only case reports or observational studies, makes thromboprophylaxis in major orthopaedic surgery a tool to be used by every clinician based on experience and case particularities (Figure 1). This review aims to briefly summarise the latest clinical data and to offer an insight into the current recommendations, that readers would find useful in daily practice. Data were identified by searches of the published literature, including MEDLINE without language restriction, along with hand search for relevant articles on reference lists, articles, and scientific proceedings.

## 2. Risk Factors for Venous Thromboembolism in Haemophilic Patients

Several factors have been incriminated in generating thrombosis in haemophilic patients: the use of central venous devices for long periods of time, replacement therapy (for deficient factors), and orthopaedic surgery. The presence of prothrombotic factors in this setting creates the perfect environment for thrombosis [8]. A patient with haemophilia scheduled to undergo orthopaedic surgery may have some risk factors that should be considered as potential triggers for a thrombotic event: increased age, previous episode of thromboembolism, varicose veins, general anaesthesia, malignancy, factor V mutation, obesity, and oral contraceptive pill (in women with von Willebrand disease) [8].

### 2.1. Central Venous Devices

Surgically inserted central venous devices (such as Port-A Cath^®^) are frequently used in patients with inherited bleeding disorders to facilitate the administration of coagulation factors and immune tolerance induction. Although they have good tolerance, they are often associated with infection, mechanical failure, and thrombosis [9]. The thrombogenic potential of the central venous devices is the result of the multiple administration of replacement therapy, which predisposes the site around the tip of the catheter to thrombus formation [10,11]. One previous study from 2001, conducted on children with haemophilia, and von Willebrand disease, showed a thrombosis rate of 50% of these devices, using contrast venography [12]. A more recent retrospective review of the same cohort, from 2011, observed a deep vein thrombosis rate of 47% of central venous devices, that occurred at a mean duration of 26 months. A total of 44% of the patients of the same population also developed post-thrombotic syndrome, but none received anticoagulation [13].

### 2.2. Concentrate Replacement Therapy

Replacement of the deficient coagulation factor is the traditional treatment for haemophilia, which may be administered intravenously on demand or as a prophylactic measure for bleeding episodes [14]. In patients with haemophilia B, who need replacement therapy with prothrombin complex concentrates (PCCs) consisting of factors II, VII, IX, and X, a high rate of thrombosis has been reported, which is linked to the activated vitamin K-dependent zymogens during manufacture [15].

**Figure 2:**
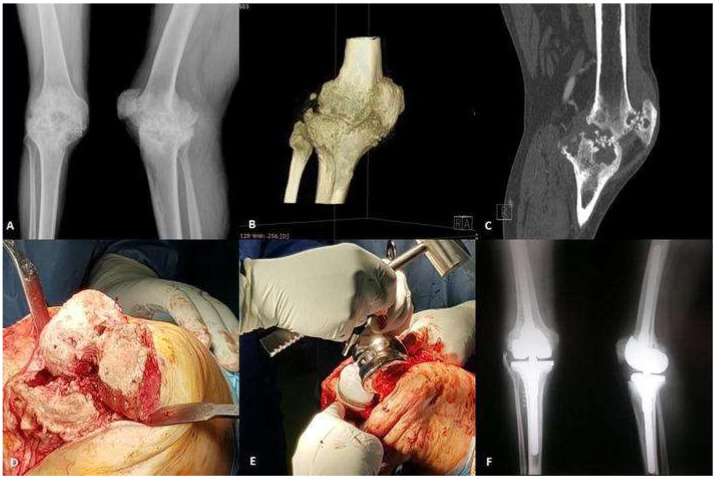
Total knee replacement in a haemophilic patient receiving concentrate replacement therapy. (**A**) Severe right gonarthrosis with bilateral genu valgum, more visible on the right side. (**B**) Typical shape and structure modifications for a haemophilic knee arthropathy (widening of the tibial plateau, tibial condyle hypertrophy, accentuation of the intercondylar incision with modification of the tibial spines and obvious rarefaction of the geodesic structure. slight collapse of the right internal tibial plateau). (**C**) periarticular osteolytic lesions with interruption of the cortex at the level of the tibial plateau and the condyles of the femur and at the level of the posterior patellar surface, with sclerotic edges and subcortical cysts.; narrowing of the femoral-tibial joint space until complete disappearance (**D**) and (**E**). Total knee arthroplasty for severe haemophilic arthropathy using a revision modular prothesis “rotating-hinge” type-intraoperative images. (**F**) Right knee total arthroplasty-intraoperatively front and lateral radiography.

While replacement therapy may seem the ideal treatment with great improvements in the quality of care (Figure 2), some patients develop neutralising antibodies, or inhibitors, against infused factor. It is estimated that approximately 30% of the patients diagnosed with severe haemophilia A develop inhibitors, along with 5% of the patients with mild and moderate forms. In patients with haemophilia B, the rates are lower, around 3% [16,17,18]. Because therapy becomes inefficient in the presence of inhibitors, bypassing agents need to be used [19]. The agents used are: aPCC (FEIBA^®^, anti-inhibitor coagulant complex, which is plasma-derived, comprising zymogens and activated coagulation factors), and recombinant-activated FVII (NovoSeven^®^). Replacement therapy with these agents may be associated with a risk of thrombosis, especially for doses higher than those recommended for aPCC (50–100 U/kg every 6–12 h), or exceeding the daily exposure limit (>200 U/kg/day), and in those with known cardiovascular risk factors [20,21]. Moreover, emicizumab (Hemlibra^®^, Roche/Genentech, South San Francisco, CA, USA) is a humanized monoclonal antibody approved for prophylactic use only in haemophilia A patients [22] with or without inhibitors, which can result in thrombotic microangiopathy and thrombosis [23]. The study published in *The New England Journal of Medicine* in 2007 conducted on 109 male participants [23], observed that this event developed after treatment with activated prothrombin complex concentrate at doses averaging more than 100 U per kilogram per day for more than 1 day during the administration of emicizumab prophylaxis.

### 2.3. Other Associated Mutations

There are a number of mutations that have been associated with a high risk of venous thromboembolism: protein C, protein S and antithrombin deficiencies, factor V Leiden, methylenetetrahydrofolate reductase (MTHFR), and β-fibrinogen genes mutations, lipoprotein (a), and prothrombin G20210A variant [24,25]. These coagulation abnormalities can modulate the clinical phenotype of haemophilia [26,27,28,29,30,31,32,33]. A case from 2000 [34] on a patient with moderate haemophilia B treated with replacement therapy reported an episode of venous thromboembolism after a total hip replacement for a hip fracture. The patient was later diagnosed with a heterozygous factor V mutation, making the author of the study suggest a preoperative screening on patients with previous thrombotic events. Associations of portal vein thrombosis, haemophilia A and FV mutation [35], deep vein thrombosis, pulmonary embolism and type III von Willebrand’s disease [36], haemophilia A, FV Leiden and cerebral infarction [10] have also been described. Moreover, according to the *European Journal of Anaesthesiology* [4,37] there were not enough data until now to justify routine screening for thrombophilia prior to surgery in patients with haemophilia.

#### 2.3.1. Subclinical Deep Vein Thrombosis

A prospective study [38] published in November 2020, including 46 patients with haemophilia, evaluated the incidence of subclinical deep vein thrombosis by Doppler ultrasound in this population. The majority of them (89.5%) underwent orthopaedic procedures under continuous infusion of clotting factor concentrates. Clinical deep vein thrombosis and pulmonary embolism were not detected, and subclinical deep vein thrombosis was present in 7.5% of cases. Only two patients were treated with a short course of low molecular weight heparin for 10–14 days. The authors of this study concluded that systematic pharmacological thromboprophylaxis in this population for the majority of patients is not necessary. The same results were published earlier, in 2010 in the *Journal Thrombosis Haemostasis* [39] which included 22 patients with haemophilia A, and B, with orthopaedic procedures that did not receive any pharmacological prophylaxis but had compression stockings as mechanical prophylaxis. No clinical deep vein thrombosis or pulmonary embolism occurred. Two cases of subclinical deep vein thrombosis in two patients with severe haemophilia A after unilateral total knee replacement were reported, which resolved spontaneously. The authors reported a distal deep vein thrombosis in a patient with haemophilia B after decompressive laminectomy for lumbar stenosis, which was treated with LMWH for two weeks at half therapeutic dosage. Because the overall incidence of thrombotic events was 10%, the authors did not recommend routine pharmacological thromboprophylaxis in haemophilic patients undergoing major orthopaedic surgery. A multicentric study from Belgium and Norway evaluated the same problem [40], including a total cohort of 65 patients-Table 2. Five cases of deep vein thrombosis in haemophilic patients treated with continuous infusion of clotting factor concentrates documented by ultrasonography imaging were observed, all were distal and not complicated by pulmonary embolism. A short course of low molecular height heparin was initiated. The calculated incidence of this event was 6%.

Another retrospective study from Japan [41] included 38 cases of total knee arthroplasty in 33 patients with haemophilia (both A and B without inhibitors) in order to assess the prevalence of lower extremity deep vein thrombosis using ultrasonography. The authors of the study did not detect signs of thrombosis (with ultrasonography being performed 2 or 3 days after surgery) in any of the cases. Mechanical prophylaxis (pneumatic compression device from day 0), and a postoperative rehabilitation programme were initiated. The low incidence for deep vein thrombosis observed in studies may be explained by the presence of relatively young patients in need of surgery, with few risk factors, early screening, and rehabilitation, and the use of mechanical prophylaxis. The choice for mechanical thromboprophylaxis was not guided by the severity of haemophilia. Home thromboprophylaxis after surgery was not mentioned by the authors of the studies mentioned [42].

#### 2.3.2. Clinical Deep Vein Thrombosis

A study [7] published in 2018 in the *Journal of Orthopaedics* which investigated the incidence of postoperative complications in 184 patients with bleeding disorders (haemophilia A, haemophilia B, FVII deficiency, and von Willebrand disease), undergoing orthopaedic surgeries found no thromboembolic event in the series investigated. A retrospective study from Beijing from 2019 [43], including 98 patients found an incidence rate of 1.02% of clinically significant venous thromboembolism. This low rate of events made the authors of the study not recommend routine thromboprophylaxis. Another retrospective study of 42 consecutive patients with haemophilia A or B, with hip or knee replacement, used mechanical thromboprophylaxis (compression stockings for up to 6 weeks after surgery for the entire cohort, and 10.5% of them also used sequential intermittent compression devices) and 2.8% of them received low-molecular weight heparin, reported an incidence of 1.4% [44] of clinical deep vein thrombosis. Interestingly enough, in a study from 2014 on 23 patients, 9% of which had a previous history of cancer, found no evidence of deep vein thrombosis at one year follow up [45]-Table 3. The severity of haemophilia did not seem to influence the decision of thromboprophylaxis. After discharge, none of the patients included received home treatment.

Markers for thrombosis

In some cases, the presence of an associated mutation may overcome the hypocoagulable state of haemophilia and predispose to thrombotic events, especially in association with other factors. Although there are case reports in literature connecting thrombotic events in patients with haemophilia with factor V Leiden mutations, the majority of cases were triggered by a concomitant risk factor (central venous catheter, factor concentrate infusion, or surgery), confirming the multifactorial etiology of thrombosis in haemophiliacs.

Thromboprophylaxis in orthopaedic patients

Prevention of venous thromboembolism can be achieved using mechanical and pharmacological methods. Mobilization, graduated compression stockings, intermittent pneumatic compression devices, and venous foot pumps, all fall in the category of mechanical methods. The major advantages of these methods are the absence of bleeding risk and laboratory monitoring, with minor side effects. Despite all benefits, there is no powerful evidence that these techniques alone can reduce the risk of death and pulmonary embolism. Additionally, there may be situations when they are difficult to implement, the compliance of the patient is reduced due to discomfort, and the need to be worn continuously pre-, intra-, and postoperatively for 72 h [45] might make them undesirable.

Unfractionated heparin, low molecular weight heparin (LMWH), adjusted dose vitamin K antagonists, synthetic pentasaccharide factor Xa inhibitor (fondaparinux), and newer oral anticoagulants, along with aspirin are the pharmacological methods used.

Administration of aspirin as a pharmacological prophylaxis method is controversial. Table 4 and Table 5 summarise the latest recommendations regarding its utilisation from the American Association of Orthopaedic Surgery (AOOS) [46], American College of Chest Physicians (ACCP) [47], The Scottish Intercollegiate Guidelines Network (SIGN) [48], and The National Institute for Health and Care Excellence (NICE). Since aspirin alone is suggested to have a role in prevention of venous thromboembolism, a large Canadian trial compared it with the oral anticoagulant agent, rivaroxaban. The Extended Venous Thromboembolism Prophylaxis Comparing Rivaroxaban to Aspirin Following Total Hip and Knee Arthroplasty (EPCAT II) [49] was a multicentre double blind noninferiority trial that included 3242 patients undergoing orthopaedic surgery. After surgery, all patients received 10 mg of rivaroxaban for 5 days, and then randomised to either 10 mg rivaroxaban daily (control group) or 81 mg of aspirin daily (intervention group) for an additional 30 days (patients undergoing total hip arthroplasty), or 9 days (patients undergoing total knee arthroplasty). Aspirin was noninferior to rivaroxaban in terms of symptomatic VTE (symptomatic PE, proximal DVT, or both), that occurred in 0.64% and 0.7% of the patients enrolled (*p* = 0.84; *p* < 0.001 for noninferiority). While the results seem to open new frontiers, the majority of the patients included had no high-risk factors for VTE such as history of VTE, recent surgery, or cancer, which makes the study hard to generalise. However, two large clinical trials are under way: The Comparative Effectiveness of Pulmonary Embolism Prevention after Hip and Knee Replacement (PEPPER) trial “https://clinicaltrials.gov/ct2/show/NCT02810704 (accessed on 13 November 2022)” and EPCAT III trial “https://clinicaltrials.gov/ct2/show/NCT04075240 (accessed on 13 November 2022)”. If the studies show promising results for the antiplatelet agent, a new and interesting pathway might be opened for other trials, including those with patients suffering from haemophilia and other bleeding disorders.

According to the 9th edition of the American College of Chest Physicians [47] (ACCP) Practice Guidelines for Prevention of Venous Thromboembolism in Orthopaedic Surgery Patients, total hip arthroplasty or total knee arthroplasty are surgical procedures that should receive prophylaxis, rather than no thrombotic prophylaxis, for a minimum of 10 to 14 days with one of the following: LMWH, fondaparinux, apixaban, dabigatran, rivaroxaban, low-dose unfractionated heparin, adjusted-dose vitamin K antagonist, (all Grade 1B), or an intermittent pneumatic compression device (Grade 1C). A significant aspect is that VTE occurs mainly after hospital discharge, making the patients with TKA or THA remain at risk weeks after surgery. Results from clinical trials have informed guideline panels to recommend extended duration of prophylaxis [50], setting the duration in the outpatient period up to 35 days from the day of the surgery [47].

Patients undergoing knee arthroscopy do not require routine pharmacologic VTE prophylaxis.

Bleeding risk

As much as thrombosis is a serious adverse event in this category, bleeding is also significant, and balancing these two entities is essential. A systematic review of randomized trials conducted by Chan et al. [8] that compared different pharmacologic regimes (apixaban, dabigatran, rivaroxaban, enoxaparin, and fondaparinux) in a total of 40,285 patients with total hip arthroplasty and total knee arthroplasty, without haemophilia, showed that symptomatic venous thromboembolism occurred in about 1% of the patients. Bleeding occurred in 0.5% to 2% of patients and raised up to 4% to 5% of them if major bleeding and clinically relevant nonmajor bleeding were combined. A prospective multicentre observational study published in 2022 in the *Journal of Thrombosis and Haemostasis* evaluated the rate of postoperative bleeding in haemophilic patients after total hip or total knee arthroplasty. A total of 131 procedures were performed, 29.8% of which were complicated by major bleeding. The incidence was higher in hip arthroplasty versus knee arthroplasty (OR 2.5, *p* = 0.05) and influenced by the presence of an inhibitor (OR 4.29, *p* = 0.04), and increased BMI (OR 4.49 and 6.09 for overweight and obese, in comparison to normal BMI, each *p* < 0.01). The authors of the study concluded that pharmacological thromboprophylaxis was not associated with bleeding risk [52]. This only shows that bleeding complications are serious events, and clinical focus should be on effectively managing these two entities.

What do guidelines say?

World Federation of Haemophilia (WFH) Guidelines for the Management of Haemophilia 3rd Edition [1].

Episodes of unprovoked venous thromboembolism are considered rare events among patients with haemophilia, a special category of patients considered to be protected. Despite these data, major surgical interventions, known to pose a risk of thrombosis (orthopaedic surgery, or abdominal surgery for cancer) seem to influence the course of the haemophilic patients. The balance between bleeding and the risk of thrombosis is fragile, which is why the current recommendations from the WFH are based on individual risk assessment. There are a number of factors that can influence the strategy applied: personal or family history of VTE, known thrombophilia, active cancer, mild haemophilia, and history of major bleeding or haemophilia B (in association with other risk factors) [37]. In cases with a high risk of thrombosis, mechanical methods for thromboprophylaxis are encouraged, with their great advantage of not elevating the bleeding risk.

The main factor that influences the balance is the administration of high doses of the deficient clotting factor. While in haemophilia A the deficient factor does not reach the high levels seen in non-haemophilia patients [53], in those with haemophilia B, there are some particularities. Currently, the WFH divides treatment for haemophilia B into two classes: pure factor IX administration, which may be plasma-derived or recombinant, and prothrombin complex concentrate (PCC) administration which consists of clotting factors II, VII, IX, and X. The same guidelines advise for pure FIX concentrates usage, as they are associated with a reduced risk of thrombosis and disseminated intravascular coagulation, compared with PCCs in the following situations: surgery, liver disease, previous thrombosis or known thrombotic tendency, concomitant use of drugs known to have thrombogenic potential, including antifibrinolytic agents, and prolonged therapy at high doses. In cases of intensive treatment, with high doses of PCCs in order to normalize FIX levels, thromboprophylaxis should be considered, according to WFH [1].

There are no consensus documents on the management of VTE in this category, but therapeutic doses of anticoagulants may be administered when deficient clotting factor levels are maintained above 30 UI/dL [54] or above 15 IU/dL [55,56], according to the same guidelines.

While in patients with haemophilia without inhibitors the use of prophylactic doses of anticoagulation is allowed, in specific situations where inhibitors appear, anticoagulants are generally contraindicated [1]. Based on clinical judgment, where the risk of haemorrhage outweighs the benefit of anticoagulation, anticoagulants are not allowed [1].

An episode of venous thromboembolism in a patient with haemophilia can be treated with high-intensity anticoagulation for the minimal duration, and with concomitant usage of replacement therapy and laboratory and clinical monitoring.

The European Society of Anaesthesiology recommends using only low molecular weight heparins, as studies with NOAC have excluded patients with bleeding disorders, and the long half-life of warfarin makes it contraindicated in this particular population [4,37].

The therapeutic approaches published in literature vary widely according to experience of the treating centre, thus there are insufficient data to compare the outcomes of different strategies.

## 3. Conclusions

Patients with haemophilia undergoing orthopaedic surgery are a category that does not benefit from evidence-based guidelines and management strategies that incorporate anticoagulation must weigh the fragile balance between haemorrhage and thrombosis. While the incidence of thrombotic events in the general population increases with age, it seems that this prothrombotic risk factor is also present in this particular category due to advances in management. Along with age, there are traditional risk factors such as diabetes, hypertension, smoking, and obesity that contribute to development of thrombi in orthopaedic surgery scenarios. The complex interaction between the prothrombotic state and tendency to bleeding poses a challenge for the treating team, that consists of haematologist, orthopaedic surgeon, anaesthesiologist, and physical rehabilitation physician. The correct management needs an individualised approach, based on risk factors, assessment of clotting factor levels, and presence of inhibitors perioperatively. The protective effect of a hypocoagulable state in haemophilia patients is not fully proven by recent data, and treatment for this disease (either prophylactically or on demand) designed to restore the haemostatic function may align thrombotic risk with that of the general population. The dilemma of whether pharmacological treatment is necessary persists, until robust data from clinical trials is available.

## Figures and Tables

**Table 1:** Severity of bleeding in relationship to clotting factor levels 1.

Severity	Clotting Factor Level	Bleeding Episodes
**Mild**	5–40 IU/dL or 5–<40% of normal	Rare spontaneous bleeding Severe bleeding occurs with major trauma or in surgical settings
**Moderate**	1–5 IU/dL or 1–5% of normal	Occasional spontaneous bleeding Prolonged bleeding occurs with minor trauma or surgery
**Severe**	<1IU/dL or <1% of normal	Spontaneous bleeding into joints or muscles, in the majority of cases in the absence of identifiable haemostatic challenge

**Table 2:** Summary of the studies reporting episodes of subclinical deep vein thrombosis.

Type of Study	Number of Patients	Number of Subclinical VTE Events	Type of Diagnosis	Thromboprophylaxis (Yes/No) Type	Side Effects	Treatment
Prospective [38]	46	5	Doppler ultrasound of lower limbs	No	No bleeding complications in 2 patients treated with LMWH	LMWH (10–14 days) at half-therapeutic dosage
Prospective [39]	22	2	Doppler ultrasound of lower limbs	Yes, mechanical (Grade 1 compression stockings)	No bleeding complication in one patient treated with LMWH	LMWH (10–14 days) At half-therapeutic dosage
Prospective, multicentric [40]	65	5 (overall incidence of 6%)	Doppler ultrasound of lower limbs	No	None mentioned	Short course of LMWH
Retrospective [41]	38	0	Doppler ultrasound of lower limbs	Yes, mechanical (pneumatic compression device from day o to day 2)	None	No

**Table 3:** Summary of the studies reporting episodes of clinical deep vein thrombosis.

Type of Study	Number of Patients	Number of Clinical VTE Events	Type of Diagnosis	Thromboprophylaxis (Y/N)-Type	Side Effects	Treatment
Retrospective [13]	184	0	-	No	No	-
Retrospective [11]	98	1	Doppler ultrasound of lower limbs	Yes, mechanical (compression stockings)	No	LMWH for 11 days
Retrospective [42]	42	1	Doppler ultrasound of lower limbs	Yes mechanical (compression stockings and intermittent compression devices) Pharmacological (Low-molecular weight heparin 2.8% of cases)	No	LMWH
Retrospective [44]	23	0	-	Yes mechanical (compression device-52%) and pharmacological (4%)	No	-

**Table 4:** Prophylaxis in Orthopaedic Surgery Patients.

Guideline	Prophylaxis Regimens	Clinical Evidence
**ACCP** [47] **(2008, 2012)**	LMWH	
	Low dose UFH	1B
	VKA	1B
	Fondaparinux	1B
	Apixaban	1B
	Dabigatran	1B
	Rivaroxaban	1B
	Aspirin	1B
	Intermittent pneumatic compression devices	1C
	Preference of LMWH to fondaparinux, apixaban, dabigatran, rivaroxaban, low dose UFH	2C
**SIGN** [48] **(2010, updated 2015)**	Preference of LMWH to VKA and aspirin	2C
	LMWH in combination with mechanical prophylaxis	A
	Fondaparinux	
	Rivaroxaban	
	Dabigatran	
	Aspirin is not recommended as a single pharmacologic agent for VTE prophylaxis	C
**AAOS** [46] **(2011)**	Use of pharmacologic agents and/or mechanical methods	Moderate
	Unclear about which prophylactic strategy (or strategies) is/are optimal or suboptimal	
	No recommendation for or against prophylactics in these patients	Inconclusive

**Table 5:** NICE [51] Guideline Recommendations for VTE Prophylaxis in Orthopaedic Patients.

NICE [51] Guideline (2018)	Indication	Duration of Treatment
**Elective total hip arthroplasty**	LMWH for 10 days followed by aspirin	10 days LMWH Further 28 days aspirin
	LMWH in combination with compression stockings (until discharge)	28 days
	Rivaroxaban Apixaban Dabigatran	>14 days
**Elective total knee arthroplasty**	Aspirin (75 or 150 mg)	14 days
	LMWH in combination with compression stockings (until discharge)	14 days
	Rivaroxaban	
	Apixaban	
	Dabigatran	>14 days

## Data Availability

Not applicable.
